# Thirteen-Week Oral Toxicity Study of HVC1 in Rats

**DOI:** 10.1155/2019/8104951

**Published:** 2019-04-11

**Authors:** Kyungjin Lee, Ho-Young Choi

**Affiliations:** Department of Herbal Pharmacology, College of Korean Medicine, Kyung Hee University, 26 Kyungheedae-ro, Dongdaemun-gu, Seoul 02447, Republic of Korea

## Abstract

Studies on the safety of herbal medicine are essential for the development of new drugs. The aim of this study was to evaluate the no-observed-adverse-effect-level (NOAEL) of HVC1 (Gamisamhwangsasim-tang, a 30% ethanol extract of a mixture of Pruni Cortex, Scutellariae Radix, Coptidis Rhizoma, and Rhei Rhizoma) and identify its target organs after oral administration to Sprague-Dawley (SD) rats repeatedly for 13 weeks. Three test groups were treated with HVC1 at a dose of either 500 (low-dose), 1,000 (middle-dose), or 2,000 (high-dose) mg/kg/day. Another group received high-dose HVC1 and was observed for 4 weeks following treatment to examine recovery from the effects of the extract. All treatment groups were compared to a vehicle control group. During the study, mortality, clinical signs, body weight changes, food consumption, abnormal lesions in the eye, urinary parameters, hematological parameters, blood coagulation time, blood biochemical parameters, changes in organ weight, gross findings, and histopathological changes were examined. No systemic toxicity related to HVC1 was observed in any group, and it was concluded that the NOAEL of HVC1 was 2,000 mg/kg/day. No target organ was identified.

## 1. Introduction

Scutellariae Radix (SR), Coptidis Rhizoma (CR), Rhei Rhizoma (RR), and Pruni Cortex (PC) have been commonly used for several thousand years in traditional Chinese, Japanese, and Korean medicine. SR has been used to treat dampness-heat, stuffiness and fullness, jaundice, high fever, thirst, hematemesis, epistaxis, and threatened abortion [1], and it has been reported to have hypotensive [2] and antihyperlipidemic effects [3]. Moreover, SR components wogonin and baicalin have vasorelaxant activities [4, 5]. CR has been used to treat dampness-heat, stuffiness and fullness, acid regurgitation, jaundice, high fever, hyperactive heart fire, inability to sleep, palpitations, hematemesis, and epistaxis [1], and it has been reported to have hypocholesterolemic activity [6] and antihyperglycemic effects. RR produces antihyperlipidemic effects [7] and has been used to treat constipation, hematemesis, epistaxis, amenorrhea, intestinal abscess, and blood stasis [1]. The herbal prescription Samhwangsasim-tang (San-Huang-Xie-Xin-Tang in Chinese), containing SR, CR, and RR, produces hypotensive effects [8] and neuroprotective effects [9, 10]. PC has been used to treat cough, urticaria, pruritus, dermatitis, asthma, and measles, and we showed that PC has a potent vasorelaxant effect [11]. These reports show that SR, CR, RR, and PC could be useful as clinical therapies for the treatment of hypertension and hyperlipidemia. Safety and efficacy studies of herbal medicines and prescriptions are necessary for the effective use of traditional medicines and new drug developments. During the process of obtaining Investigational New Drug (IND) approval of these herbal medicines, the Ministry of Food and Drug Safety of Korea demands efficacy data and toxicity data including acute, subacute (4-week), and repeated dose (13-week) oral toxicity. Therefore, we prepared an herbal extract (Gamisamhwangsasim-tang, abbreviated HVC1) of SR, CR, RR, and PC and investigated the hypotensive and hypolipidemic effects of this preparation and the results have already been reported [12–15]. In addition, we have also evaluated acute and subacute toxicity studies and reported safety results [15]. This study was conducted to assess the cumulative toxicity of HVC1 when orally administered once daily to SD rats for a period of 13 weeks at doses of 500, 1,000, and 2,000 mg/kg/day. The reversibility of any effects during a 4-week recovery phase was also examined.

## 2. Materials and Methods

### 2.1. Compliance with Ethical and Procedural Standards

This study was performed at Korea Conformity Laboratories (KCL, Incheon, Korea) with the approval of KCL's Institutional Animal Care and Use Committees (IA14-00150). The procedures used in this study were in compliance with KCL's ethical standards, in accordance with Good Laboratory Practice (GLP) of the Ministry of Food and Drug Safety (MFDS, No. 2013-40), and in line with the applicable guidelines for repeated-dose 90-day oral toxicity studies in rodents (OECD guideline No. 408) and the testing guidelines for safety evaluation of drug (MFDS guideline No. 2014-06).

### 2.2. Preparation of HVC1

A decoction of HVC1 was produced by Kolmar Korea Co., Ltd. (Seojeong, Korea) from a mixture of chopped crude herbs (Table 1). Professor Hocheol Kim of Kyung Hee University identified the materials. The herbal mixture (20 kg) was extracted with 30% ethanol for 2 h in a reflux apparatus. After reflux and filtration, the solvent was removed under reduced pressure at 80–95°C to yield 4.6 kg of crude extract (1 g extract = about 4.35 g raw/dried herb).

### 2.3. Test Substance and Dosing Solution

HVC1 was dissolved in distilled water and dosing solutions were prepared daily. The dose volume was 10 mL/kg/day for all animals. To maintain a constant dose, the dose volume was adjusted to the latest recorded body weight for each individual rat.

### 2.4. High-Performance Liquid Chromatography (HPLC) Conditions

The HPLC analysis was performed at room temperature, at a flow rate of 0.5 mL/min, and for a duration of 100 min using the same equipment used in the previous study [16]. The injection volume was 10 *μ*L. The mobile phase consisted of 0.1% formic acid (A) and acetonitrile (HPLC grade, J.T. Baker®, Avantor Performance Materials, Inc., Center Valley, PA, USA) (B) in a ratio specified by the following binary gradient with linear interpolation: 0 min, 20% B; 60 min, 30% B; 70 min, 60% B; and 100 min, 70% B. The column eluent was monitored at 250 nm.

### 2.5. Animals

Male and female pathogen-free SD rats (approximately 5-week-old) were obtained from Orientbio Inc. (Sungnam, Gyeonggi province, Korea). On arrival, the animals were examined for signs of ill health and were allowed to acclimatize for 6 days prior to start of the study. One day before the start of oral administration, the animals were divided into 4 main groups and 2 recovery groups according to body weight. The rats were housed in stainless steel cages with a barrier system to control the light-dark cycle (08–20 h), air exchange (10–15 changes/h), temperature (23.1 ± 1.0°C), and relative humidity (54.2 ± 5.6 %) during the study. Animals were provided Teklad certified global 18% protein rodent diet (Harlan Co., Ltd., Madison, WI, USA) and filtered municipal tap water. Food and water were provided* ad libitum*.

### 2.6. Experimental Design

#### 2.6.1. Experimental Groups and Administration

In previous studies using rats, the effective dose of HVC1 was 250 mg/kg/day. We calculated the pharmacologically active dose in humans according to guidelines on converting animal doses to human-equivalent doses [17]: 250 mg/kg/day/6.17 (conversion factor for rats)/10 (safety factor) × 60 (mean weight of a person in kg) = 243.1 mg/kg/day. Therefore, the recommended daily dose of HVC1 for humans is 250 mg/person/day. In a previous dose selection study (repeated dosing of 0, 500, 1000, or 2000 mg/kg/day HVC1 for 4 weeks), treatment-related toxicity was not observed. Therefore, in the present 13-week study, the dose range of 0, 500, 1,000, and 2000 mg/kg/day HVC1 was chosen.

The study included 4 main groups with 10 males and 10 females in each group and 2 recovery groups with 5 males and 5 females in each group. The main groups were a vehicle (distilled water) control group and 3 treatment groups receiving 500, 1,000, or 2,000 mg/kg/day HVC1 for 13 weeks. The recovery groups (a vehicle-treated control group and a group treated with high-dose (2,000 mg/kg/day) HVC1) received treatment for 13 weeks, followed by 4 weeks without further treatment. The test substances and vehicle were orally administered once daily into the stomach by gavage (using a stainless steel catheter attached to a disposable syringe).

#### 2.6.2. Clinical Sign Observation and Body Weight

During the course of the experiment, all animals were observed daily for clinical signs. Body weight and food consumption were measured weekly just before dosing and on the day of necropsy.

#### 2.6.3. Ophthalmoscopy and Urinalysis

Ophthalmoscopic examinations of all rats were conducted prior to the start of the treatment and prior to necropsy by using an ophthalmoscope (Vantage plus, Keeler Ltd., Windsor, UK) and a digital fundus camera (Genesis, Kowa Co., Ltd., Nagoya, Japan).

Urine was collected for urinalysis from 5 rats in each group prior to necropsy. Urinalysis was performed to assess urine volume, specific gravity, pH, glucose, bilirubin, ketone bodies, protein, urobilinogen, nitrates, leukocytes, cast, occult blood, red blood cells, white blood cells, and epithelial cells using a urine analyzer (CliniTek 50, Siemens, Munich, Germany).

#### 2.6.4. Hematology and Serum Biochemistry

Blood samples were collected from an abdominal artery and analyzed to determine white blood cell count, red blood cell count, hemoglobin concentration, hematocrit, neutrophil count, lymphocyte count, monocyte count, eosinophil count, basophil count, large unstained cell count, reticulocyte count, platelet count, percentage of neutrophils, percentage of lymphocytes, percentage of monocytes, percentage of eosinophils, percentage of basophils, percentage of large unstained cells, mean corpuscular volume, mean corpuscular hemoglobin concentration, red cell width distribution, and mean platelet volume using an automatic hematological analyzer (ADVIA 2120, Siemens, Munich, Germany). In addition, blood samples were treated with 3.2% sodium citrate and analyzed for prothrombin time and activated partial thromboplastin time using an automatic coagulation time meter (ACL 7000, Instrumentation Laboratory, Bedford, MA, USA).

Serum biochemistry analysis was performed to assess the levels of aspartate aminotransferase, alanine aminotransferase, alkaline phosphatase, gamma(*γ*)-glutamyl transferase, lactate dehydrogenase, blood urea nitrogen, creatinine, glucose, total cholesterol, total protein, creatine phosphokinase, albumin, total bilirubin, triglycerides, uric acid, calcium, inorganic phosphorus, chloride, magnesium, sodium, and potassium, as well as albumin/globulin ratio, by using an automatic serum biochemical analyzer (Hitachi 7180, Hitachi, Tokyo, Japan).

#### 2.6.5. Gross Observation and Measurement of Organ Weights

After exsanguination from the abdominal aorta, macroscopic examination of the full external surface, cranial cavity, and organs of the thoracic and abdominal cavities was performed. The absolute weights of the spleen, liver, adrenal glands, kidney, heart, lung, brain, pituitary, thymus, testis, prostate, ovary, and uterus were measured, and their relative organ weights (organ-to-body weight ratios) were calculated.

#### 2.6.6. Histopathology

Histopathological analysis of the brain, pituitary gland, heart, lung, liver, kidney, urinary bladder, mesenteric lymph node, thymus, spleen, parathyroid gland, adrenal gland, esophagus, aorta, spinal cord, sciatic nerve, skeletal muscle, skin, mammary gland, eye, cecum, colon, rectum, femur, sternum, trachea, tongue, prostate gland, testes, epididymis, pancreas, salivary gland, submandibular lymph node, thyroid gland, stomach, duodenum, jejunum, ileum, seminal vesicle, ovary, uterus, and vagina was performed. All organs were preserved in 10% neutral buffer formalin except the testes (preserved in Bouin's solution) and eyes (preserved in Davison's solution).

### 2.7. Statistical Analysis

Statistical analyses were performed using SPSS v.12.0 statistical analysis software (IBM Corp., Armonk, NY, USA). One-way analysis of variance (ANOVA) followed by Dunnett's multiple comparison test was used for the statistical analyses of body weight, food consumption, urine volume, hematology, plasma coagulation, serum biochemistry, and organ weight. The chi-square test and Kruskal-Wallis test were used for the analyses of specific gravity, glucose, protein, leukocyte, ketone, occult blood, bilirubin, nitrite, and urobilinogen. Results were considered statistically significant for* p* values less than 0.05.

## 3. Results

### 3.1. Calibration Curve and Limits of Detection and Quantification

Calibration curves were calculated for the 9 reference compounds using linear regression derived from the peak area. The regression equations (correlation coefficient, *R*^2^) of the reference compounds are shown in Table 2. The calibration curves had good linearity.

### 3.2. HPLC Analysis of HVC1

**Figure 1:**
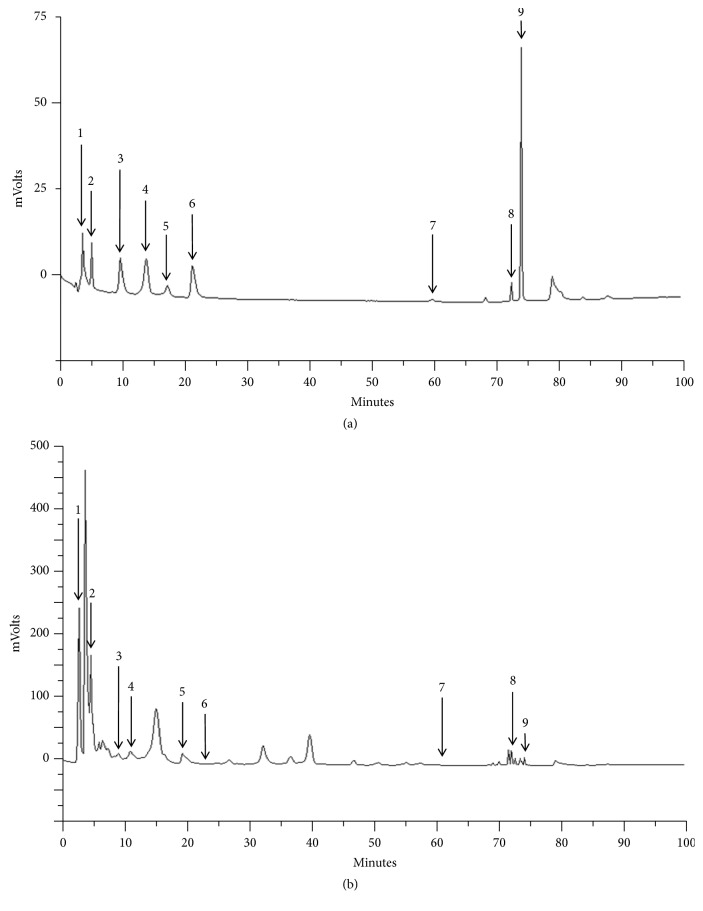
HPL chromatogram of standard mixtures (a) and HVC1 (b). Compounds: 1, sennoside A; 2, genistein-7-O-*β*-glucopyranoside; 3, coptisine; 4, baicalin; 5, prunetin-5-O-*β*-glucopyranoside; 6, berberine; 7, baicalein; 8, wogonin; and 9, prunetin.

Nine compounds in HVC1 were measured via HPLC (Figure 1). The contents of sennoside A, genistein-7-O-*β*-glucopyranoside, coptisine, baicalin, prunetin-5-O-*β*-glucopyranoside, berberine, baicalein, wogonin, and prunetin in HVC1 were 14.12 ± 3.81, 1.71 ± 0.08, 0.85 ± 0.01, 25.89 ± 0.00, 16.89 ± 3.09, 3.58 ± 0.45, 0.40 ± 0.00, 7.13 ± 0.03, and 0.71 ± 0.00 mg/g, respectively.

### 3.3. Mortality, Clinical Signs, and Body Weight

**Figure 2:**
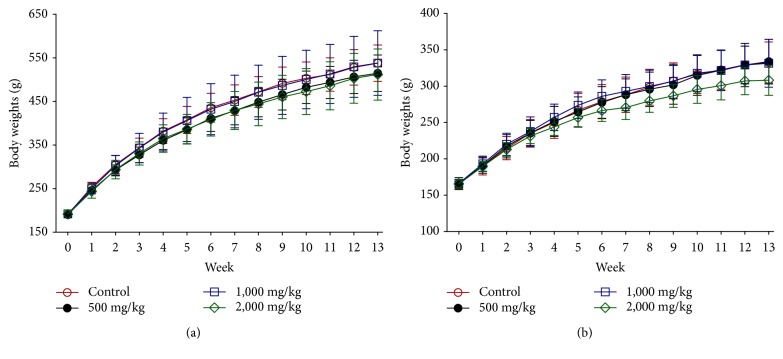
Body weight of male (a) and female (b) rats treated with 0 (control), 500, 1,000, or 2,000 mg/kg HVC1 for 13 weeks. Values are expressed as mean ± SD.

Throughout the 13-week study period, no signs of ill health or mortality related to HVC1 treatment were observed. There were no significant differences in body weight between the control and HVC1-treated groups (Figure 2). There were no significant differences in feed intake between the control and HVC1-treated groups (data not shown). There were no significant differences in clinical observations, body weight, and feed intake during the recovery period (data not shown).

### 3.4. Ophthalmoscopy and Urinalysis

Ophthalmoscopic examination did not reveal changes after HVC1 treatment (data not shown).

Urinalysis showed significant differences in glucose level, ketone body level, and leukocyte count between the control and HVC1-treated male rats. Significant differences in ketone body level were observed between the control and HVC1-treated female rats. No other significant differences between the control and HVC1-treated male and female groups were observed (Table 3).

### 3.5. Hematology and Serum Biochemistry

In male rats, white blood cell levels significantly increased in the groups treated with 1,000 and 2,000 mg/kg/day HVC1 (*p* < 0.05), and the percentage of lymphocytes in the group treated with 2,000 mg/kg/day HVC1 also significantly increased (*p* < 0.01). The mean corpuscular hemoglobin level in the group treated with 500 mg/kg/day HVC1 and the percentage of neutrophils in the group treated with 2,000 mg/kg/day HVC1 significantly decreased (*p* < 0.05) in comparison with those of the control group. No other significant differences in hematological and plasma coagulation values were observed between the control and HVC1-treated male and female groups (Tables 4 and 5).

In male rats treated with 2,000 mg/kg/day HVC1, levels of uric acid (*p* < 0.05), inorganic phosphorus (*p* < 0.01), magnesium (*p* < 0.05), and potassium (*p* < 0.01) significantly increased. No other significant differences in serum biochemical values were observed between the control and HVC1-treated male and female groups (Tables 6 and 7).

### 3.6. Gross Observations and Organ Weights

No treatment-related changes in gross findings were observed in any of the treated animals. Absolute spleen weight in the male rats treated with 500 or 2,000 mg/kg/day HVC1 significantly decreased (*p* < 0.05), whereas absolute spleen weight significantly increased in the female rats treated with 1,000 mg/kg/day HVC1, and absolute kidney weight of both kidneys significantly increased in the female rats treated with 1,000 or 2,000 mg/kg/day HVC1 (*p* < 0.05) (Table 8). The relative weight of the left kidney in the male rats treated with 1,000 or 2,000 mg/kg/day HVC1 significantly increased (*p* < 0.05). The relative weight of the spleen in the female rats treated with 1,000 mg/kg/day HVC1 (*p* < 0.05) and the relative weight of both the kidneys in the female rats treated with 1,000 or 2,000 mg/kg/day HVC1 significantly increased (*p* < 0.01) (Table 9). No other significant differences in absolute or relative organ weights were observed between the control and HVC1-treated male and female groups (data not shown).

### 3.7. Histopathological Findings

No abnormal histopathological changes were observed in any animals (data not shown).

## 4. Discussion

In the present study, no signs of ill health or mortality were observed after oral administration of HVC1. Although salivation was sporadically observed in the male and female rats treated with 1,000 and 2,000 mg/kg/day, it was concluded that this symptom was temporarily stimulated by administration and did not have any toxicological significance. There were no significant differences in body weight and feed intake between the control and HVC1-treated groups.

Ophthalmoscopy revealed no abnormal lesions in any of the animals examined. The urinalysis showed significant differences in glucose, ketone body level, and leukocyte level in HVC1-treated male rats and in ketone body level in HVC1-treated female rats, in comparison with the control group. The color of the urine changed in a dose-dependent manner in animals of both sexes in all dosing groups and tended to have a reddish color (amber, orange, or dark red). The change in urine color was thought to be caused by the test substance. No changes were observed in biochemical parameters related to the kidneys, such as blood urea nitrogen and creatinine levels, and histopathological examination revealed no kidney-related abnormalities. In addition, no differences in urine color were observed during the 4-week recovery period, and changes in urine color were thus not considered a toxic result of HVC1 treatment.

According to hematological testing, the male rats in HVC1-treated groups showed increased white blood cell count and lymphocyte percentage, as well as decreased mean corpuscular hemoglobin level and neutrophil percentage. However, these changes were within the range of natural biological variation and were not observed during the recovery period. No other significant differences in hematological parameters and plasma coagulation values were observed.

Analysis of serum biochemistry revealed increased levels of uric acid, inorganic phosphorus, magnesium, and potassium in the male rats treated with 2,000 mg/kg HVC1. However, these changes were within the range of natural biological variation. These differences were not observed during the recovery period.

The absolute spleen weights in male rats decreased, while the absolute and relative weights of the spleen and both kidneys in females, as well as the relative weight of the left kidney in males, increased. However, these changes in organ weight were not dose-dependent and were not observed during the recovery period, and no spleen- or kidney-related abnormalities were observed in the biochemical and histopathological analyses. No abnormal histopathological changes related to HVC1 oral administration were observed in any animals.

## 5. Conclusion

In this study, no systemic toxicity related to HVC1 was observed in any of the primary treatment or recovery groups after repeated dosing for 13 weeks. Therefore, the NOAEL (no-observed-adverse-effect-level) of HVC1 was established as 2,000 mg/kg/day, and no target organ of HVC1 toxicity was identified. This result suggested that there are no toxicity and side effects from repeated administration of HVC1. For the clinical trial, we have obtained IND approval from this result and previous hypolipidemia efficacy results and toxicity results in Korea. We are going to conduct phase II clinical trials. Like this, many studies are currently being carried out actively to discover new efficacies of traditional herbal prescriptions and to develop new prescriptions in Korea. If many clinical trials based on ethnopharmacological studies are carried out, it is expected that many new drugs using herbal medicine could be developed.

## Figures and Tables

**Table 1:** Preparation of HVC1.

Herb	Used part	Amount (kg)	Company of purchase	Source
Pruni Cortex	Stem bark	6.7	DWD	Korea
Rhei Rhizoma	Root	6.7	DWD	China
Coptidis Rhizoma	Root	3.3	DYH	China
Scutellariae Radix	Root	3.3	DYH	Korea
Total amount		20.0		
Extract yield		4.6		

DWD: Dongwoodang Co., Ltd. (Yeongcheon city, Kyungpook, Republic of Korea).

DYH: Dong Yang Herb Co., Ltd. (Seoul, Republic of Korea).

**Table 2:** Calibration parameters of HPLC analysis for the 9 tested compounds.

Compound	Regression equation	R^2^
1	*y* = 4699.07x − 32464.02	0.9950
2	*y* = 17643.82x − 1925.72	0.9998
3	*y* = 54479.75x − 146134.56	0.9996
4	*y* = 29573.31x − 72978.81	0.9999
5	*y* = 55396.74x + 6805.61	0.9995
6	*y* = 48543.21x − 108301.56	0.9999
7	*y* = 17086.68x − 57907.43	0.9994
8	*y* = 8381.04x − 14106.11	0.9999
9	*y* = 104880.25x − 190133.26	0.9997

Compounds: 1, sennoside A; 2, genistein-7-O-*β*-glucopyranoside; 3, coptisine; 4, baicalin; 5, prunetin-5-O-*β*-glucopyranoside; 6, berberine; 7, baicalein; 8, wogonin; 9, prunetin.

**Table 3:** Urinalysis results of HVC1-treated male and female rats.

Sex	Test item (grade)	Treatment group (mg/kg/day)			
		Control	500	1,000	2,000
Males	Glucose (0–4)	0.0 ± 0.0	0.0 ± 0.0	0.4 ± 0.5	1.2 ± 0.4^*∗*^
	Ketone body (0–4)	0.6 ± 0.9	1.8 ± 0.4^*∗∗*^	2.0 ± 0.0^*∗∗*^	2.6 ± 0.5^*∗∗*^
	Leukocyte (0–4)	0.8 ± 0.4	1.0 ± 1.7	4.0 ± 0.0^*∗∗*^	4.0 ± 0.0^*∗∗*^

Females	Ketone body (0–4)	0.0 ± 0.0	1.2 ± 0.8^*∗*^	1.6 ± 0.5^*∗*^	1.6 ± 0.5^*∗*^
	Leukocyte (0–4)	0.0 ± 0.0	1.6 ± 2.2^*∗∗*^	0.8 ± 1.8^*∗∗*^	1.6 ± 2.2^*∗∗*^

Values are expressed as mean ± SD (*n *= 5). ^*∗*^*p* < 0.05, ^*∗∗*^*p* < 0.01 compared with the control group.

Grades: 0 (negative), 1 (trace), 1+, 2, 2+, 3, 3+, 4.

**Table 4:** Hematological and plasma coagulation values of HVC1-treated male rats.

Test item (unit)	Treatment group (mg/kg/day)			
	Control	500	1,000	2,000
WBC (K/*μ*L)	9.86 ± 1.77	9.01 ± 2.41	11.78 ± 2.54^*∗*^	11.71 ± 3.27^*∗*^
Neutrophil (K/*μ*L)	1.64 ± 0.68	1.47 ± 0.42	1.92 ± 0.49	1.34 ± 0.38
Lymphocyte (K/*μ*L)	7.83 ± 1.65	7.12 ± 2.05	9.35 ± 2.13	9.92 ± 2.82
Monocyte (K/*μ*L)	0.21 ± 0.07	0.24 ± 0.08	0.29 ± 0.08	0.25 ± 0.10
Eosinophil (K/*μ*L)	0.08 ± 0.03	0.07 ± 0.02	0.08 ± 0.03	0.07 ± 0.04
Basophil (K/*μ*L)	0.00 ± 0.00	0.01 ± 0.01	0.01 ± 0.00	0.01 ± 0.01
LUC (K/*μ*L)	0.10 ± 0.04	0.10 ± 0.04	0.14 ± 0.08	0.13 ± 0.06
NEP (%)	16.8 ± 6.2	16.8 ± 3.6	16.4 ± 3.9	11.5 ± 1.5^*∗*^
LYP (%)	79.2 ± 5.8	78.6 ± 3.3	79.2 ± 3.9	84.6 ± 2.0^*∗∗*^
MOP (%)	2.2 ± 0.9	2.6 ± 0.6	2.4 ± 0.5	2.1 ± 0.6
EOP (%)	0.8 ± 0.3	0.8 ± 0.3	0.7 ± 0.2	0.6 ± 0.3
BAP (%)	0.0 ± 0.1	0.1 ± 0.0	0.1 ± 0.0	0.1 ± 0.1
LUP (%)	1.0 ± 0.4	1.1 ± 0.4	1.2 ± 0.5	1.0 ± 0.3
RBC (K/*μ*L)	8.81 ± 0.28	8.80 ± 0.44	8.72 ± 0.42	8.69 ± 0.43
Hemoglobin (g/dL)	15.6 ± 0.2	15.2 ± 0.6	15.2 ± 0.6	15.5 ± 0.8
Hematocrit (%)	45.1 ± 1.7	44.2 ± 1.8	44.1 ± 1.8	45.3 ± 2.7
MCV (fL)	51.3 ± 1.8	50.2 ± 1.7	50.6 ± 1.5	52.1 ± 1.6
MCH (pg)	17.8 ± 0.5	17.2 ± 0.6^*∗*^	17.4 ± 0.4	17.9 ± 0.5
MCHC (g/dL)	34.7 ± 1.0	34.3 ± 0.3	34.4 ± 0.5	34.3 ± 0.7
RDW (%)	12.8 ± 1.0	12.2 ± 0.9	12.6 ± 0.5	12.6 ± 0.5
Platelet (K/*μ*L)	1068 ± 119	1015 ± 102	1111 ± 104	1108 ± 113
MPV (fL)	6.4 ± 1.3	6.6 ± 1.4	6.5 ± 1.4	6.5 ± 1.5
Reticulocyte (%)	2.05 ± 0.80	1.60 ± 0.59	1.85 ± 0.55	1.94 ± 0.33
PT (sec)	9.89 ± 0.71	9.60 ± 0.63	9.39 ± 0.21	9.40 ± 1.01
APTT (sec)	13.7 ± 2.7	16.0 ± 0.9	15.9 ± 1.3	15.7 ± 1.9

WBC: white blood cell; LUC: large unstained cell; NEP: percent of neutrophil; LYP: percent of lymphocyte; MOP: percent of monocyte; EOP: percent of eosinophil; BAP: percent of basophil; LUP: percent of large unstained cell; RBC: red blood cell; MCV: mean corpuscular volume; MCH: mean corpuscular hemoglobin; MCHC: mean corpuscular hemoglobin concentration; RDW: red cell distribution width; MPV: mean platelet volume; PT: prothrombin time; APTT: active partial thromboplastin time. Values are expressed as mean ± SD (*n *= 10). ^*∗*^*p* < 0.05, ^*∗∗*^*p* < 0.05 compared with the control group.

**Table 5:** Hematological and plasma coagulation values of HVC1-treated female rats.

Test item (unit)	Treatment group (mg/kg/day)			
	Control	500	1,000	2,000
WBC (K/*μ*L)	5.47 ± 2.32	6.19 ± 2.46	5.94 ± 1.46	7.17 ± 3.34
Neutrophil (K/*μ*L)	0.80 ± 0.37	0.79 ± 0.31	0.72 ± 0.15	0.87 ± 0.37
Lymphocyte (K/*μ*L)	4.39 ± 2.03	5.10 ± 2.17	4.95 ± 1.42	5.97 ± 2.91
Monocyte (K/*μ*L)	0.14 ± 0.07	0.15 ± 0.06	0.15 ± 0.10	0.16 ± 0.07
Eosinophil (K/*μ*L)	0.08 ± 0.05	0.08 ± 0.04	0.07 ± 0.03	0.08 ± 0.05
Basophil (K/*μ*L)	0.00 ± 0.00	0.00 ± 0.00	0.00 ± 0.00	0.00 ± 0.00
LUC (K/*μ*L)	0.06 ± 0.06	0.06 ± 0.04	0.05 ± 0.03	0.08 ± 0.05
NEP (%)	15.5 ± 7.2	13.3 ± 4.3	12.8 ± 4.6	12.6 ± 2.3
LYP (%)	79.3 ± 7.3	81.8 ± 4.4	82.6 ± 4.4	82.5 ± 3.6
MOP (%)	2.5 ± 0.7	2.5 ± 0.7	2.5 ± 1.1	2.3 ± 0.9
EOP (%)	1.6 ± 1.1	1.3 ± 0.6	1.2 ± 0.7	1.4 ± 1.3
BAP (%)	0.1 ± 0.1	0.1 ± 0.0	0.1 ± 0.1	0.1 ± 0.0
LUP (%)	1.1 ± 0.7	1.0 ± 0.4	0.8 ± 0.3	1.1 ± 0.3
RBC (K/*μ*L)	7.85 ± 0.38	8.01 ± 0.39	7.78 ± 0.64	8.14 ± 0.32
Hemoglobin (g/dL)	14.7 ± 0.7	15.2 ± 0.7	14.9 ± 0.7	15.2 ± 0.6
Hematocrit (%)	43.3 ± 2.7	44.7 ± 2.7	43.3 ± 3.0	44.5 ± 0.9
MCV (fL)	55.2 ± 2.3	55.8 ± 1.9	55.7 ± 1.8	54.7 ± 1.4
MCH (pg)	18.8 ± 0.6	19.0 ± 0.5	19.2 ± 1.0	18.7 ± 0.4
MCHC (g/dL)	34.0 ± 1.1	34.0 ± 1.2	34.4 ± 1.6	34.1 ± 1.0
RDW (%)	11.5 ± 0.5	11.6 ± 0.4	11.6 ± 1.3	11.2 ± 0.4
Platelet (K/*μ*L)	1097 ± 138	1038 ± 165	965 ± 367	1122 ± 131
MPV (fL)	8.6 ± 1.0	8.5 ± 0.7	8.9 ± 1.3	8.6 ± 0.7
Reticulocyte (%)	2.01 ± 0.26	2.03 ± 0.26	2.76 ± 2.31	1.90 ± 0.39
PT (sec)	8.78 ± 0.61	8.87 ± 0.47	8.84 ± 0.41	8.73 ± 0.53
APTT (sec)	14.9 ± 3.5	16.2 ± 1.3	15.1 ± 2.5	15.9 ± 1.2

WBC: white blood cell; LUC: large unstained cell; NEP: percent of neutrophil; LYP: percent of lymphocyte; MOP: percent of monocyte; EOP: percent of eosinophil; BAP: percent of basophil; LUP: percent of large unstained cell; RBC: red blood cell; MCV: mean corpuscular volume; MCH: mean corpuscular hemoglobin; MCHC: mean corpuscular hemoglobin concentration; RDW: red cell distribution width; MPV: mean platelet volume; PT: prothrombin time; APTT: active partial thromboplastin time. Values are expressed as mean ± SD (*n *= 10).

**Table 6:** Serum biochemical values in HVC1-treated male rats.

Test item (unit)	Treatment group (mg/kg/day)			
	Control	500	1,000	2,000
AST (IU/L)	113 ± 22	113 ± 34	109 ± 24	125 ± 47
ALT (IU/L)	30 ± 4	31 ± 8	32 ± 10	41 ± 25
ALP (IU/L)	275 ± 39	280 ± 51	263 ± 38	233 ± 37
GGT (IU/L)	0.1 ± 0.3	0.1 ± 0.3	0.1 ± 0.3	0.1 ± 0.3
Lactate dehydrogenase (IU/L)	1441 ± 585	1295 ± 651	1123 ± 351	1164 ± 340
Blood urea nitrogen (mg/dL)	18.0 ± 1.8	16.6 ± 2.0	17.2 ± 2.1	15.7 ± 1.1
Creatinine (mg/dL)	1.08 ± 0.84	1.29 ± 0.76	1.04 ± 0.50	0.87 ± 0.64
Glucose (mg/dL)	179 ± 20	180 ± 27	192 ± 24	176 ± 26
Total cholesterol (mg/dL)	64 ± 9	57 ± 7	59 ± 7	59 ± 13
Total protein (g/dL)	6.2 ± 0.2	6.0 ± 0.2	6.1 ± 0.2	6.0 ± 0.4
Creatine phosphokinase (U/L)	644 ± 261	576 ± 284	480 ± 166	522 ± 147
Albumin (g/dL)	2.1 ± 0.1	2.0 ± 0.1	2.0 ± 0.1	2.1 ± 0.2
Total bilirubin (mg/dL)	0.03 ± 0.01	0.04 ± 0.02	0.03 ± 0.03	0.06 ± 0.04
Albumin/Globulin ratio	0.51 ± 0.03	0.52 ± 0.04	0.49 ± 0.03	0.53 ± 0.04
Triglyceride (mg/dL)	48 ± 19	39 ± 18	39 ± 13	36 ± 13
Uric acid (mg/dL)	1.9 ± 0.5	1.9 ± 0.4	2.2 ± 0.3	2.6^*∗*^ ± 0.7
Calcium (mg/dL)	9.3 ± 0.4	9.4 ± 0.4	9.6 ± 0.4	9.8 ± 0.5
Inorganic phosphorus (mg/dL)	9.4 ± 0.6	9.2 ± 0.6	9.8 ± 0.6	10.7^*∗∗*^ ± 1.1
Chloride (mmol/L)	106 ± 1	107 ± 2	107 ± 2	107 ± 1
Magnesium (mg/dL)	2.6 ± 0.2	2.6 ± 0.2	2.8 ± 0.2	2.9^*∗*^ ± 0.3
Sodium (mmol/L)	144 ± 1	145 ± 1	144 ± 2	144 ± 1
Potassium (mmol/L)	5.0 ± 0.7	5.1 ± 0.6	5.4 ± 0.3	6.3^*∗∗*^ ± 1.0

AST: aspartate aminotransferase; ALT: alanine aminotransferase; ALP: alkaline phosphatase; GGT: gamma(*γ*)-glutamyl transferase. Values are expressed as mean ± SD (*n *= 10).^*∗*^*p* < 0.05, ^*∗∗*^*p* < 0.05 compared with the control group.

**Table 7:** Serum biochemical values in HVC1-treated female rats.

Test item (unit)	Treatment group (mg/kg/day)			
	Control	500	1,000	2,000
AST (IU/L)	153 ± 85	138 ± 57	136 ± 71	121 ± 27
ALT (IU/L)	44 ± 25	40 ± 21	34 ± 22	33 ± 14
ALP (IU/L)	110 ± 21	135 ± 30	114 ± 27	136 ± 49
GGT (IU/L)	0.0 ± 0.0	0.3 ± 0.5	0.0 ± 0.0	0.0 ± 0.0
Lactate dehydrogenase (IU/L)	1307 ± 848	1381 ± 838	1300 ± 598	1340 ± 407
Blood urea nitrogen (mg/dL)	20.6 ± 3.6	18.6 ± 2.1	21.9 ± 6.2	21.9 ± 6.5
Creatinine (mg/dL)	0.69 ± 0.08	0.69 ± 0.09	0.70 ± 0.17	0.72 ± 0.15
Glucose (mg/dL)	154 ± 29	167 ± 35	167 ± 41	152 ± 33
Total cholesterol (mg/dL)	85 ± 18	82 ± 15	81 ± 14	79 ± 19
Total protein (g/dL)	7.1 ± 0.4	6.9 ± 0.3	7.0 ± 0.4	7.0 ± 0.3
Creatine phosphokinase (U/L)	571 ± 347	592 ± 351	624 ± 323	542 ± 137
Albumin (g/dL)	2.9 ± 0.2	2.8 ± 0.2	2.9 ± 0.3	2.8 ± 0.2
Total bilirubin (mg/dL)	0.10 ± 0.04	0.08 ± 0.03	0.12 ± 0.13	0.07 ± 0.02
Albumin/Globulin ratio	0.69 ± 0.02	0.67 ± 0.05	0.68 ± 0.05	0.68 ± 0.04
Triglyceride (mg/dL)	49 ± 30	56 ± 31	45 ± 21	40 ± 25
Uric acid (mg/dL)	1.8 ± 0.4	1.7 ± 0.4	1.9 ± 0.4	1.7 ± 0.4
Calcium (mg/dL)	10.1 ± 0.5	10.1 ± 0.4	10.3 ± 0.6	10.2 ± 0.5
Inorganic phosphorus (mg/dL)	8.3 ± 1.3	8.4 ± 0.7	9.0 ± 0.8	9.3 ± 1.0
Chloride (mmol/L)	106 ± 3	105 ± 1	106 ± 1	105 ± 2
Magnesium (mg/dL)	2.9 ± 0.2	2.9 ± 0.2	2.9 ± 0.3	3.1 ± 0.2
Sodium (mmol/L)	143 ± 1	143 ± 1	143 ± 1	143 ± 1
Potassium (mmol/L)	5.2 ± 0.9	5.2 ± 0.6	5.6 ± 0.9	5.6 ± 0.5

AST: aspartate aminotransferase; ALT: alanine aminotransferase; ALP: alkaline phosphatase; GGT: gamma(*γ*)-glutamyl transferase. Values are expressed as mean ± SD (*n *= 10).

**Table 8:** Absolute organ weights (g) in HVC1-treated male and female rats.

Sex	Organ	Treatment group (mg/kg/day)			
		Control	500	1,000	2,000
Males	Spleen	0.991 ± 0.180	0.805 ± 0.124^*∗*^	0.924 ± 0.147	0.842 ± 0.115^*∗*^
Females	Spleen	0.580 ± 0.090	0.565 ± 0.042	0.812 ± 0.412^*∗*^	0.598 ± 0.086
	Kidney (left)	0.950 ± 0.105	0.969 ± 0.096	1.095 ± 0.154^*∗*^	1.076 ± 0.113^*∗*^
	Kidney (right)	0.983 ± 0.125	1.001 ± 0.010	1.126 ± 0.157^*∗*^	1.118 ± 0.110^*∗*^

Values are expressed as mean ± SD (*n *= 10). ^*∗*^*p* < 0.05 compared with the control group.

**Table 9:** Relative organ weights (g) in HVC1-treated male and female rats.

Sex	Organ	Treatment group (mg/kg/day)			
		Control	500	1,000	2,000
Males	Kidney (left)	0.297 ± 0.034	0.299 ± 0.023	0.325 ± 0.026^*∗*^	0.327 ± 0.030^*∗*^
Females	Spleen	0.186 ± 0.029	0.182 ± 0.020	0.261 ± 0.127^*∗*^	0.208 ± 0.031
	Kidney (left)	0.305 ± 0.041	0.310 ± 0.027	0.352 ± 0.043^*∗∗*^	0.374 ± 0.038^*∗∗*^
	Kidney (right)	0.315 ± 0.045	0.320 ± 0.022	0.363 ± 0.052^*∗∗*^	0.389 ± 0.039^*∗∗*^

Values are expressed as mean ± SD (*n *= 10). ^*∗*^*p* < 0.05, ^*∗∗*^*p* < 0.01 compared with the control group.

## Data Availability

The data used to support the findings of this study are available from the corresponding author upon request.

## Abbreviations

HVC1: A 30% ethanol extract of a mixture comprising Pruni Cortex, Scutellariae Radix, Coptidis Rhizoma, and Rhei Rhizoma.
SHR: Spontaneous hypertensive rat.
